# Evaluation of Apoptosis-related Genes and Hormone Secretion Profiles Using Three Dimensional Culture System of Human Testicular Organoids

**DOI:** 10.31661/gmj.v12i.2805

**Published:** 2023-08-26

**Authors:** Aghbibi Nikmahzar, Farnaz Khadivi, Morteza Koruji, Mehrdad Jahanshahi, Masoomeh Dehghan Tarazjani, Maryam Shabani, Yasaman Abbasi, Mehdi Abbasi

**Affiliations:** ^1^ Department of Anatomy, School of Medicine, Tehran University of Medical Sciences, Tehran, Iran; ^2^ Department of Anatomy, School of Medicine, Shahrekord University of Medical Sciences, Shahrekord, Iran; ^3^ Stem Cell and Regenerative Center, Iran University of Medical Sciences, Tehran, Iran; ^4^ Department of Anatomy, School of Medicine, Iran University of Medical Sciences, Tehran, Iran; ^5^ Neuroscience Research Center, Department of Anatomy, Faculty of Medicine, Golestan University of Medical Sciences, Gorgan, Iran; ^6^ Vali-E-Asr Reproductive Research Center, Family Research Institute, Tehran University of Medical Sciences, Tehran, Iran; ^7^ Program in Neuroscience, Center to Advance Chronic Pain Research, Department of Neural and Pain Sciences, School of Dentistry, University of Maryland, Baltimore, MD, United States

**Keywords:** Spermatogonial Stem Cells, Organoid Culture, Apoptosis, 3D Culture

## Abstract

Background: In reproductive biology, testicular organoids can be used to treat infertility and to study testicular development and spermatogonial stem cells (SSCs) differentiation. Generating organoid from primary cells is challenging. In this study, testicular organoids were created using human primary testicular cells and evaluated the apoptotic gene expression and hormone secretion profiles of the organoids. Materials and Methods: Primary human testicular cells were isolated using 2-step enzymatic digestion from three brain-dead donors. Immunocytochemistry and flow cytometry analyses were performed to confirm human SSCs. Isolated cells were cultured in three experimental groups: control group (2 dimensional (2D)), group 1 (organoid culture after 2D culture), and group 2 (organoid culture immediately after enzymatic digestion). Testicular organoids were cultured in DMEM/F-12 media supplemented with follicle-stimulating hormone (FSH) and fetal bovine serum (FBS) for four weeks. After 24 hours and four weeks of culture, reverse transcription quantitative real-time PCR (RT-qPCR) was used to investigate the relative expression of apoptotic genes (caspase 3, 9, Bax, and Bcl-2). At 24 hours, two weeks, and four weeks after culture, enzyme-linked immunoassay (ELISA) was used to determine the testosterone and inhibin B concentrations. Light microscopy and toluidine blue staining were also used for morphological analysis. Results: RT-qPCR results revealed that pro-apoptotic (caspase 3, 9, Bax) gene expression levels were highest in group 2 after 24 h and four weeks of culture. In contrast, the expression level of Bcl-2 (anti-apoptotic) was lower in group 2 compared to other groups. The hormone secretion levels decreased in a time-dependent manner during the cultivation. According to morphological evaluations, testicular organoids are compact, spherical structures with two to three elongated cells organized along their border. Conclusion: Our findings revealed that the testicular organoid culture system maintained hormonal secretory abilities, demonstrating the function of Sertoli and Leydig cells in the absence of testis-specific environments.

## Introduction

Remarkable advances in pediatric cancer therapy have significantly increased the life expectancy of cancer survivors by up to 80% [1][2]. Gonadotoxic chemotherapy and radiotherapy treatment could damage extremely spermatogonial stem cells (SSCs) and can lead to male infertility [3]. Before gonadotoxic medications, the most effective strategy for maintaining male fertility in adult men and young teenagers is sperm cryopreservation [4]. Because spermatogenesis does not begin until puberty, this strategy cannot be used on prepubertal boys.

The only way to preserve a childs’ fertility is to cryopreserve testicular tissue or SSCs prior to cancer treatment [5]. Recently, in vitro spermatogenesis using two and three-dimensional (2D and 3D) culture techniques has received considerable attention to obtain functional sperm and restore fertility after cryopreservation in these patients [6][7]. Compared to 2D conditions, 3D culture methods can provide an ideal microenvironment for SSCs differentiation and proliferation. They can create a suitable condition like the testis microenvironment [8].

The organ-culture method, microfluidic systems, air-liquid interface, 3D printing, and organoids are examples of 3D culture systems [9]. Organoids are novel strategies generated from tissue-specific stem cells and provide the investigation of the developmental mechanisms in an in vitro system [10][11]. These structures have been used to create a variety of tissues, including the intestine [12], brain [13], liver [14], prostate [15], etc., that conduct distinct stages of organ development or particular function of the organ [16]. Testicular organoids appear as a proper model for research because they can be easily manipulated and rapidly reorganized from testicular cells [9][17].

Testicular organoids can investigate testicular development, reorganization, and interactions between different cell populations in the testis niche. This is a helpful approach for identifying the unknown factors involved in the survival, proliferation, and differentiation of SSCs [9][11][17]. Previous experiments have demonstrated that testicular organoid culture systems can create appropriate conditions that promote SSCs survival and differentiation [18][19]. Testicular organoids’ can be generated using different techniques, and there is no generally accepted protocol for testicular organoid culture. Three-layer gradient system, hanging drop technique, and decellularized testicular tissue fragments were utilized to generate testicular organoids [17][18][19][20].

Previous testicular organoid studies have only focused on animal models [19][21] and pluripotent or immortalized Leydig and Sertoli cells [18].

The organoids generation from immortalized cells could not be transferred to humans. The production of human testicular organoids using primary human testicular cells has received little attention due to the limited proliferation ability and complex challenges in testicular cell culture. Immortal cells are different from primary cultured cells. Since they have undesired genetic alterations and different molecular structures, which makes them immortal.

The results of primary cell culture may be obtained in human experiments because they are directly taken from tissues using optimized enzymatic digestion [22]. Recently, researchers generated human testicular organoids using first-trimester human embryonic gonadal cells in a three-layer gradient method. Hormone production and differentiation of Sertoli and Leydig cells were examined after one week of 3D culture [20].

The pool of testicular tissue within the SSCs must be preserved. Apoptosis is a permanent event in early development and the mature testis. The SSCs pool in the testes must be preserved.

Any defects in consecutive mitosis and meiosis divisions during spermatogenesis can result in the progression of apoptosis and the elimination of abnormal cells [23].

The death of testicular germ cells involves two apoptotic pathways: the internal system, also known as the mitochondrial mechanism, and the extrinsic pathway, also known as the death receptor [24]. Death receptors, including Fas (CD95L), tumor necrosis factor (TNF), and others, activate the initiator caspase 8 in the extrinsic pathway. Bcl-2 catalyzes intrinsic or mitochondrial reactions. Caspase 3 is activated by both internal and extrinsic caspase activation, which increases the probability of apoptosis [23][25]. For the first time, we investigated the apoptotic gene expression levels in human testicular cells being cultured in an organoid culture system. Also, the levels of inhibin B and testosterone were analyzed to assess the functioning of human testicular organoids.

## Materials and Methods

1. Sample Collection and Enzymatic Digestion

Testicular tissues were donated by three brain-dead donors from Sina Hospital, a part of Tehran University of Medical Science. Consent was obtained from the patients’ relatives by the organ Procurement Unit of Sina Hospital before utilizing the tests in this study. This study was approved by the Ethics Committee of the Tehran University of Medical Sciences (IR.TUMS.VCR. REC.1398.450). For the testicular cell isolation, a two-step enzymatic digestion procedure was used according to the Baert et al. protocol [26,27]. After enzymatic digestion, the cell suspensions were filtered using a cell strainer with a 40 m mesh size to obtain a suspension of single cells. Hemocytometer was used for counting obtained cells, and the viability rate was calculated using 0.04 percent Trypan blue (Sigma-Aldrich).

2. Culture and Proliferation of Human SSCs

The procedure was performed according to the previous study on the propagation of human primary testicular cell [28]. The somatic cells elimination was performed using the differential plating method. Floating cells were then collected and cultured at a density of 15000 to 20000 cells/cm2 in 25 cm2 flasks in DMEM/F12 containing 5% fetal bovine serum (FBS; Gibco, Paisley, Uk), 10 ng/mL glial cell line-derived neurotrophic factor (GDNF; G1401, Sigma-Aldrich), and 10 ng/mL fibroblast growth factor (bFGF; F3685, Sigma-Aldrich), 10 ng/ml leukemia inhibitory factor (LIF; L5283, Sigma Aldrich), and 5% knock-out serum replacement (KSR; Invitrogen, USA). For 3 weeks flasks were incubated at 35°C and the culture media was changed every two to three days. At the end of the 2D culture period, the cells were trypsinized and used for organoid culture and further analysis. Trypsin-EDTA (0.25%) was utilized to trypsinize the cultured cells and then used for organoid culture and further analysis.

3. Formation and Culture of Testicular Organoids

In this study, we performed a 3D organoid culture system using the Pendergraft et al. method [18]. Immediately following enzymatic digestion and 3 weeks of human SSC 2D culture, isolated cells were employed for 3D organoid culture. A 1:1 ratio of matrigel (Matrigel; P/N 356231, Corning, Tewksbury, MA, USA) and DMEM/F12 containing 10% FBS was used to suspend the cells. A droplet with a density of 10000 cells/20 μL was used for the hanging drop culture technique. Organoids were transferred onto 24-well plates containing DMEM/F12 supplemented by 10% FBS and 2.5 × 10−5 IU recombinant follicle-stimulating hormone (FSH, Sigma-Aldrich), 100 ng/mL recombinant human stem cell factor (SCF, Sigma-Aldrich. Louis, MO, USA). After 2 days of incubation at 37 °C in 5% CO2, organoids were cultured in 3 groups: Control group: 2D culture of human SSCs for 4 weeks. Group 1: 2D culture of human SSCs and organoid culture for 4 weeks. Group 2: organoid culture immediately after enzymatic digestion for 4 weeks.

4. SSCs Confirmation Tests

4.1. RT-PCR Analysis

After enzymatic digestion, RT-PCR was used to analyze the relative expression of VASA, OCT4, PLZF (human germ cell-specific genes), Sertoli and Leydig cell-specific genes (Vimentin and CYP11A1). Three replicates of each evaluation were performed. Utilizing a qiazol reagent (Qiagen, Hilden, Germany) and according to the recommended guidelines, total RNA was extracted. spectrophotometry apparatus (Eppendorf, Hamburg, Germany) was used to assess the purity and agarose gel electrophoresis (1.5% w/v agarose/TEA) was performed using the protocol of Mushtaq et al. to analyze the integrity of the total RNA [29].

**Table:** Table 1. The Designed Primers for RT-PCR and RT-qPCR Analyses

	Gene	Type and sequences	Product size (bp)	Annealing temperature (◦C)
1	PLZF	Forward:CGGGACTTTGTGCGATGTG Reverse:GCGGTGGAAGAGGATCTCAA	106	59
2	SYCP3	Forward : GGAAGGAGTTGGAGTTGACAT Reverse : ATCCCACTGCTGAAACAAAGTC	190	59
3	PRM2	Forward:ATGCTGCCGCCTGTGGAT Reverse:GCCAAGAGGAGCAAGGGC	125	61
4	Oct4	Forward:CTGGGTTGATCCTCGGACCT Reverse:CACAGAACTCATACGGCGGG	128	60
5	Vimentin	Forward : CGTGAATACCAAGACCTGCTC Reverse CTGCTCTCCTCGCCTTCC	89	59
6	CYP11A1	Forward : CTGCATCTTCAGTCGTCTGTCC Reverse : GGTGACCACTGAGAACCCATTC	83	61
7	BAX	Forward:GCGACTGATGTCCCTGTCTC Reverse:AAAGATGGTCACGGTCTGCC	77	60
8	Bcl-2	Forward:TGGTGGGAGCTTGCATCAC Reverse:GCATATTTGTTTGGGGCAGGC	77	62
9	VASA	Forward:ATCAACCCTCATCTGTCTTCC- Reverse:TATTACACTCACCACCATCTCT	196	60
10	Caspase 9	F: GAGGACACAGGCCAGGACATG R: CACTGGTCTGGGTGTTTCCGG	156	62
11	Caspase 3	F:CCGTGGTACAGAACTGGACTG R: ACAAGAAGTCGGCCTCCACT	95	60
12	GAPDH	Forward:GCACCGTCAAGGCTGAGAAC Reverse:ATGGTGGTGAAGACGCCAGT	142	61

The genomic DNA contamination was eliminated using DNase I (Fermentas, Waltham, MA, USA). Complementary DNA (cDNA) was generated using extracted RNA (1 g), random hexamers, oligo (dT), and a cDNA synthesis kit (Fermentas, Waltham, MA, USA).

Table-1 shows the primers utilized in the current study. When the cDNA was synthesized, PCR products were subjected to 1.5 percent (w/v) agarose gel electrophoresis, and the UV gel doc system was used to monitor the gels’ appearance.

4.2. SSCs Purity and Flow Cytometry Analyasis

For the calculation of the SSCs’ purity percentage, flow cytometry analysis was used. Following the enzymatic digestion and 3 weeks 2D culture flow cytometric analysis using a PLZF marker was accomplished by applying standard procedures. Paraformaldehyde (PFA; Sigma-Aldrich) containing 4% and 0.4% Triton X100 was used to fix and permeabilize the primary testicular cells. Following permeabilization, 15 μl of the anti-PLZF primary antibody (anti-PLZF antibody, 1:100, ab104854, Abcam, Cambridge, MA, USA) with 106 cells were incubated overnight at room temperature.

The cells were washed in PBS, and then at 4 °C anti-mouse FITC conjugated secondary antibody (1:180; ab97022, Abcam, USA) was added. A Beckman Coulter flow cytometer (Partec AG, CH-4144 Arlesheim, Switzerland) equipped with a 15-mW argon-ion laser and 488 nm excitation wavelength was used.

4.3. Immunocytochemistry Assay

Immunocytochemistry analyses were used to identify the human Sertoli cells marker (Vimentin) and SSCs specific markers (PLZF, GFRα-1), following the 3 weeks of 2D culture. For this purpose, 4 % PFA (fixation) and 0.5 % Triton X100 (permeabilization) were added respectively. Five percent bovine serum albumin (BSA; Sigma-Aldrich) was added to inhibit the non-specific binding regions.

The fixed cells were treated with anti-GFRα-1 (sc-28319, Santa Cruz Biotechnology, USA) and anti-PLZF (sc-271546, Santa Cruz Biotechnology, USA) primary antibodies at 1:100 concentrations and 37 °C for 2 h.and was visualized using DAPI (Sigma Aldrich) staining. The negative control has no primary antibody. Fluorescence pictures were acquired using a fluorescence microscope (Olympus BX51TRF, Tokyo, Japan) and a camera.

4.5. Quantitative PCR (q-PCR) Evaluation

In the experimental groups, after 24 hours and 4 weeks of culture, apoptotic gene expression was assessed using qPCR and the comparative CT approach. Total RNA extraction from 2D culture, performed using a qiazol (Qiagen, Hilden, Germany) reagent, and in organoid cultures was performed by the Da Silva et al. protocol (30). PCR gene expression was quantitatively assessed by an Applied BioSystems (Applied BioSystems, Foster City, USA) RT-qPCR equipment using the SYBR Green Premix Ex Taq Kit (Tli RNaseH Plus). As an internal control, the gene glyceraldehyde-3-phosphate dehydrogenase (GAPDH) was chosen. Table-1 shows the primers used to determine gene expression levels by qPCR. All amplification reactions were carried out in triplicate. The comparative CT method (ΔΔCT) was used to assess the relative gene expression.

4.6. Histological Analysis

After four weeks of cultivation, a morphological analysis of human testis organoids was performed using light microscopy. The human testis organoids were fixed in 4% PFA for 1 hour, dehydrated through increasing ethanol concentrations, and xylene cleaning was followed by paraffin embedding and sectioning to a thickness of 5 μm. Toluidine blue staining was used to stain the prepared sections and histological analysis was carried out by light microscopy (Olympus, Japan).

4.7. Statistical Analysis

For data analysis, SPSS software (version 16.0, Armonk, NY, USA) was used. All statistical analysis results were reported as mean ± standard deviation (SD). One-way analysis of variance (ANOVA) and Tukey’s test were used for apoptotic gene expression analysis, and two-way ANOVA and Bonferroni post hoc test were performed for hormone secretion profile data analysis. P values of <0.05 were considered statistically significant.

## Results

Evaluation of Human SSCs Viability

**Figure-1:**
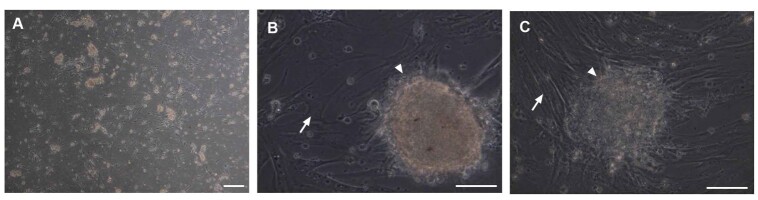
Figure-1. Human SSCs morphologies during 2D cultures. (A-B): After three weeks of culture, the arrow indicated the Sertoli cells, and the arrowhead displayed the SSCs colonies. All scale bars=500μm.

Trypan blue was utilized to measure the vitality of human cultured cells after enzymatic isolation and after three weeks of culture. After enzymatic digestion, the viability percentage of freshly acquired human testicular cells was more than 70%. The viability rate of obtained human SSCs colonies increased above 88% after 2D culture for three weeks. One week after the testicular cell suspension culture, small colonies of SSCs appeared on the surface of the Sertoli cells (Figure-1A), and SSC colonies proliferated and became larger after 3 weeks and became large (Figure-1B and 1C).

Human SSCs Colonies and Sertoli Cells Identification by Immunocytochemistry

**Figure-2:**
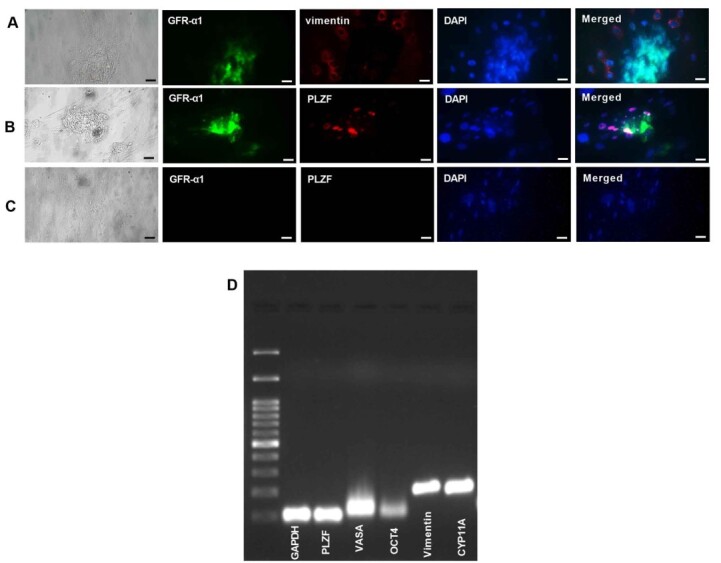
Figure-2. Immunocytochemistry and RT-PCR analyzes for SSCs characterization. (A-D): Double immunostaining against GFR-α1, Vimentin, and PLZF in human SSCs 2D culture after 3 weeks. (A): GFR-α1 and Vimentin double immunostaining. (B): GFR-α1 and PLZF double immunostaining. (C): Negative control. All scale bars=20 μm. (D): RT-PCR images. The results for SSCs (VASA, OCT4, and PLZF), Sertoli cells (Vimentin), and Leydig cells (CYP11A) detection at the RNA level, after enzymatic digestion. GAPDH was used as the internal control.

GFRα-1 and PLZF proteins Expression as markers of undifferentiated SSCs was detected in obtained human SSCs colonies after 2D culture of human testicular cells. Also, after 3 weeks of 2D culture, Sertoli cells express the specific marker Vimentin according to the immunocytochemistry results (Figure-2A-C).

Human SSCs Characterization by RT-PCR

The RT-PCR data showed the expression of PLZF, VASA, OCT4 (human SSCs markers), Vimentin (Sertoli cells marker), and CYP11A1 (Leydig cells marker) after enzymatic digestion (Figure-2D).

Purification Percentage of Human SSCs by Flow Cytometry Analysis

**Figure-3:**
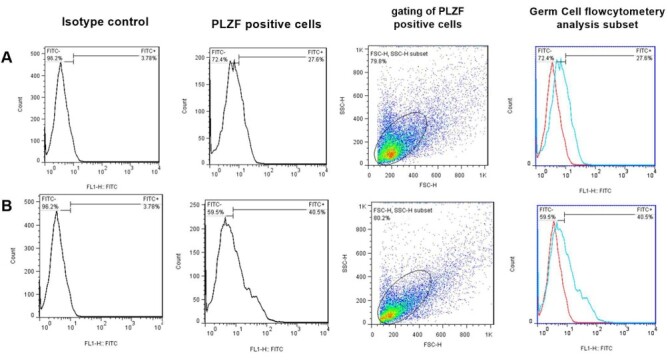
Figure-3. Flow cytometry analyses for PLZF marker in primary testicular cells. A: Following two-step enzymatic isolation at day 0. B: After 3 weeks 2D culture.

Flow cytometric analysis was performed after enzymatic digestion and 3 weeks of 2D culture using a PLZF marker. Data showed that after enzymatic digestion, 27.6% of testicular cell suspension was possess the PLZF marker (Figure-3A). The number of SSCs increased significantly after three weeks of 2D culture, reaching 40.5 % (Figure-3B).

Morphological Assessment of Human Testicular Organoids

**Figure-4:**
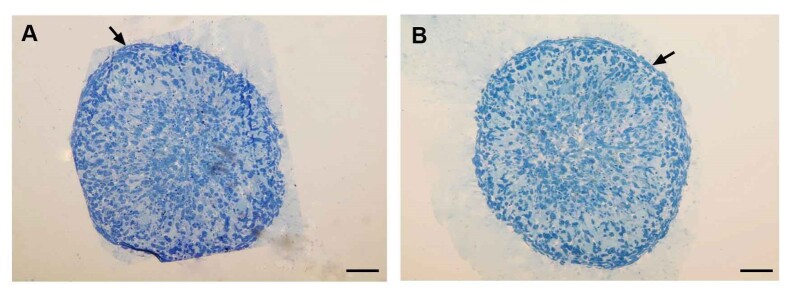
Figure-4. Morphological assessment of testicular organoids after 4 weeks culture. All groups had similar morphology and cell arrangement, with higher compaction in organoid edges. Arrowheads indicate the elongated cells in border of organoids. (A-B): Toluidine blue staining of semi-thin sections for morphological evaluation of testicular organoids. (A) group 1 , (B) group 2. Scale bars:100 μm.

Toluidine blue staining showed close morphological similarity in groups 1 and 2. Human testicular organoids displayed a compact structure after 4 weeks of culture. This compaction was pronounced in both groups at the boundary of organoids; two to three layers of compact cells were seen within the border of organoids. Arrowheads indicate the elongated cells (Figure-4).

Apoptosis-related Genes Expression in Human Testicular Organoids

**Figure-5:**
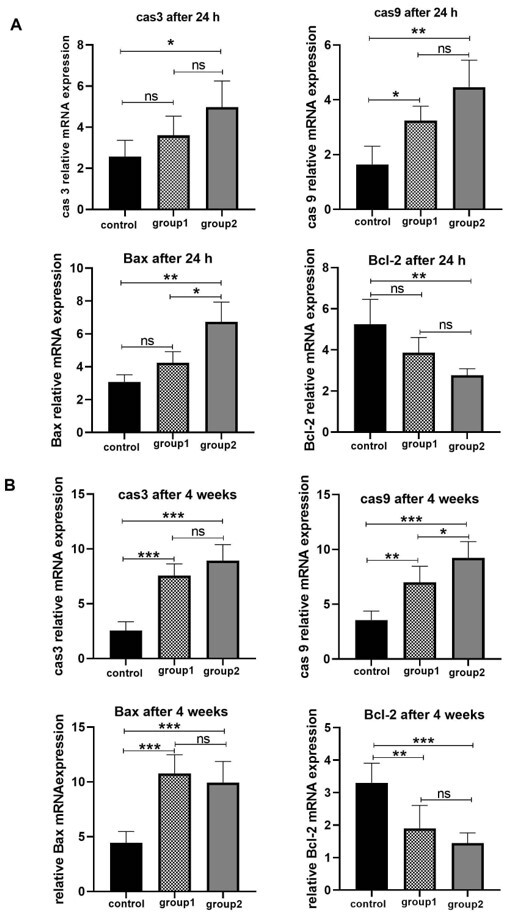
Figure-5. Apoptosis genes qPCR results. (A): Expression rates of cas 3, cas 9, Bax and Bcl-2 after 24h. (B): Expression rates of cas 3, cas 9, Bax and Bcl-2 after 4 weeks culturing in three groups. *: P>0.05; **: P<0.005; ***: P<0.0005. Data are shown as means ± SD (n=3).

The apoptosis gene expression in SSCs was investigated by examining apoptosis genes, including caspase 3, caspase 9, Bax, and Bcl-2. Our findings demonstrated that after 24 hours of culture, group 2 had greater expression levels of caspase 3, caspase 9, and Bax than the other groups (P≤0.05). There was no significant difference in caspase 3 and 9 relative expressions between group 1 and group 2 (P≥0.05). Data showed that after 24 hours of culture, Bcl-2 (anti-apoptotic) expression was higher in the control group compared to experimental group 2 (P≤0.05). No significant difference was found between test experimental groups 1 and 2, as well as between the control group and group 1. (P≥0.05). The relative expression level of pro-apoptotic genes (caspase 3, 9, and Bax) after 4 weeks of culture was upregulated in group 1 and group 2 compared to the control (P≤0.05). Based on the RT- qPCR findings, the highest level of Bcl-2 gene expression was observed in the control (P≤0.05). In contrast, Groups 1 and 2 did not indicate any significant difference (P≥0.05 , Figure-5).

Hormone Secretory Profile in Organoids

**Figure-6:**
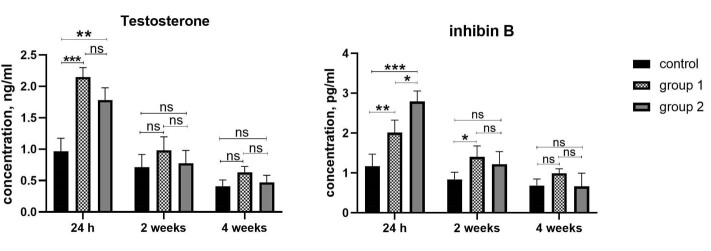
Figure-6. Testosterone and inhibin B secretory profile. Testosterone and inhibin B in the medium were measured after 24 h, 2 weeks and 4 weeks of culturing. *: P<0.05; **: P<0.005; ***: P<0.0005. The data is shown as means ± SDs (n=3).

The presence of hormones (testosterone and inhibin B) in the culture medium is essential to demonstrate the functionality of human testicular organoids. The presence of active Leydig and Sertoli cells in organoids was confirmed by the detection of testosterone and inhibin B, respectively. The results showed that human testicular organoids are functionally active and they can secrete hormones. Testosterone and inhibin B levels in culture supernatants after 24 hours, two weeks, and four weeks of culture showed that at 24h after organoid culture, testosterone concentration in group 1 had the highest level compared to the other groups (P ≤ 0.0001). The difference between groups 1 and 2 was insignificant following 24 h of culture (P>0.05). We did not observe a significant difference in testosterone levels between the 3 groups, after 4 weeks of culture. The concentration of inhibin B was higher in group 2 compared to group 1 and the control group, also the difference between groups 1 and 2 was remarkable (P<0.05). After two weeks of culture, there was a significant difference in the amount of inhibin B between group 2 and other groups. Similar to testosterone, we did not observe significant differences between groups after 4 weeks of culture (Figure-6).

Our data showed that testosterone and inhibin B levels were reduced during two weeks of organoid culture compared to 24 hours of culture in all three groups. This reduction in hormone levels continues until the end of the culture period (fourth week) (Figure-6). The findings demonstrate that despite the culture medium containing the FSH, the hormone secretion ability of adult human testicular organoids time-dependently decreased.

## Discussion

In this study, we produced testicular organoids from primary human testicular cells. The morphology, hormone secretion profiles, and apoptotic gene expression were examined under two different conditions: after 2D culture and immediately after enzymatic digestion. Cell arrangements and morphological characteristics were similar in groups of human testicular organoids. The formation of spheroid structures with elongated compact cells at the boundaries was demonstrated. It was discovered that pro-apoptotic gene levels increased while Bcl-2, an anti-apoptotic gene, decreased after 4 weeks of culture in the enzymatic digestion group (group 2) compared to other groups.

Concerning recent advances in human organoid culture, drug efficacy assessment may now be assessed in 3D primary cultures. Unfortunately, similar progress has not yet been achieved in the testicular organoids. Testicular organoids are helpful approaches to assess in vitro spermatogenesis, development, and physiology of the human testis [31].

The apoptotic pathways (extrinsic and intrinsic) are active in the testicular cells. In the mitochondrial or intrinsic pathway, Bax is transferred from the cytoplasm to the mitochondria. Cytochrome c is released into the cytoplasm, stimulating programmed cell death via apoptosis in this pathway. Members of the Bcl-2 protein contribute to the intrinsic pathway through its interaction with Bax [23]. The Fas ligand activates the Fas protein on the cell membranes, activating the extrinsic pathway, commonly known as the death receptor mechanism [32].

It is well known that the intrinsic apoptosis pathway regulates the population of testicular germ cells. According to previous studies, failure to regulate apoptosis during the first phase of spermatogenesis resulted in increased spermatogonial cells in mice lacking the Bax gene [32][33].

After 24 hours of culture, group 2 showed higher levels of apoptotic gene expression than the other groups. This increase resulted in the enzymatic digestion of human testicular tissue by collagenase and hyaluronidase. Expression of apoptotic genes decreased in group 1 using 2D culture after enzymatic digestion. After four weeks of organoid culture, it was found that the expression of pro-apoptotic genes in group 2 was increased compared to other groups, and the expression of anti-apoptotic genes was decreased. It was observed that group 2 had higher levels of pro-apoptotic and lower rates of anti-apoptotic gene expression levels compared to the other groups.

Similar to our experimental results, in testicular tissue culture, Bax expression levels increased while Bcl-2 expression levels decreased [34][35]. Also, the previous study’s findings demonstrated that the percentage of cells that undergoes apoptosis increased in human testicular tissue culture. On the other hand, selective germ cell apoptosis is necessary for spermatogenesis [35].

Our results revealed that the concentration of hormones decreased in the second week of the existence of FSH in the culture media, and the level of hormones decrease gradually during the study. The physiological concentration of gonadotropins did not affect the generation of testosterone or Inhibin B. Presence of inhibin B and testosterone in the conditioning media suggested the functionality of Leydig and Sertoli cells during the culture. In agreement with our results, another study demonstrated that the secretion of inhibin B and testosterone was reduced in testicular organoids despite the presence of gonadotropins in the culture medium. The culture of mature testicular cells leads to failure in stimulating gonadotropin response [17].

The histological evaluations revealed that human primary testicular cells were structurally transformed into spheroidal organoids. No evidence of necrosis in testicular organoids was observed by toluidine blue staining. Oval or round-shaped cells were detected in the central compartment of the organoids, while spindle- or elongated fibroblast-like cells were found in the outer part. According to our results, spindle-shaped cells might be peritubular myoid cells. Although, further investigation is required to be done. Similar to our research, the expression of the α-SMA marker as the marker of myoid cells was reported in elongated peritubular-like cells at the border of human testicular cell clusters in the 2D culture of human testicular cells [36].

We created spheroid-shaped testicular organoids using the hanging drop approach. The interaction between surface tension and gravity field is the basis of the hanging-drop technique. Spheroid-shaped organoids help stimulate the cell to ECM interactions, resulting in chemical and cellular gradients forming similar to in vivo conditions [37]. According to the previous experiment, the advantage of the hanging drop technique is the ability to easily manipulate testicular organoids and control the size and organization of cells [18].

Testicular organoids could provide insight into the processes that explain normal spermatogenesis and underlying diseases. The 3D culture systems that are currently available are only appropriate for quick assessments [38]. The missing piece in the treatment of non-obstructive azoospermia and male fertility preservation in the clinic is a technique that permits in vitro spermatogenesis [39].

## Conclusion

Generation of organoids from primary human testicular cells in a 3D microenvironment is a novel approach that enables cell-cell interactions, cell polarization, ECM production, and cell-specific gene expression. Spherical testicular organoids are easy to manipulate and investigate. Also, the functioning of Leydig and Sertoli cells could be maintained in the testicular organoids. Testicular organoids can be suggested for in vitro normal spermatogenesis, drug toxicity research, and future clinical applications.

## Acknowledgments

A grant (98-02-30-42456) from the Tehran University of Medical Sciences supported this research. The findings of this paper were included in a Ph.D. thesis.

## Conflict of Interest

The authors declare that they have no conﬂict of interest.
